# The bisbenzimide compound ridinilazole has antiviral activity against vaccinia and mpox viruses

**DOI:** 10.1099/jgv.0.002363

**Published:** 2026-11-02

**Authors:** Misbah El-Jesr, Lucy Eke, Blair L. Strang, Carlos Maluquer de Motes, Rebecca P. Sumner

**Affiliations:** 1 Department of Microbial Sciences, University of Surrey, Guildford GU2 7XH, UK; 2 Institute of Infection & Immunity, St George’s School of Health and Medical Sciences, City St George’s, University of London, London, SW19 0RE, UK; 3 Department of Infectious Diseases, King’s College London, London SE1 9RT, UK

**Keywords:** antiviral, bisbenzimide, mpox, orthopoxvirus, ridinilazole, vaccinia virus

## Abstract

Mpox remains a significant global public health threat, highlighting the need for new antiviral therapies. Ridinilazole is a symmetric bisbenzimide developed for the treatment of *Clostridium difficile* infection that has recently been shown to inhibit human cytomegalovirus. Here, we investigated its antiviral activity against orthopoxviruses (OPXV). Ridinilazole potently inhibited vaccinia virus replication in primary human foreskin fibroblasts, with an IC_50_ of 21 nM and minimal cytotoxicity. Importantly, ridinilazole also reduced infectious monkeypox virus (the causative agent of mpox) yields in primary fibroblasts by more than 150-fold. Mechanistic studies demonstrated that ridinilazole did not affect early viral gene expression or viral DNA replication, but significantly reduced the expression of late viral genes at both transcript and protein levels. These findings identify ridinilazole as an effective inhibitor of OPXV replication and support its potential repurposing as a novel therapeutic for mpox.

## Introduction

Orthopoxviruses (OPXV) are a family of large dsDNA viruses that replicate exclusively in the cytoplasm. OPXV have a broad host range and infect a variety of rodent species (natural reservoir) and incidental hosts. Some, such as variola virus (VARV), the causative agent of smallpox, and monkeypox virus (MPXV), the causative agent of mpox, possess the ability to infect humans and cause disease, with VARV becoming human-specific. Smallpox was eradicated in 1980 following a highly successful vaccination campaign using the related OPXV vaccinia virus (VACV) as a vaccine. However, cessation of vaccination since then has led to waning anti-OPXV immunity in the global human population and increased opportunity for the emergence of zoonotic OPXV. Indeed, whilst historically confined to regions of central and western Africa with limited human-to-human transmission, mpox infections in humans have increased significantly over recent years resulting in multiple independent outbreaks [1]. The first mpox outbreak started in 2022 caused by a clade IIb strain (lineage B.1) deriving from endemic clade IIb lineage A strains in West Africa [2, 3]. This virus showed an unprecedented ability to transmit human-to-human, particularly via sexual contact and resulted in a global outbreak with >100,000 reported cases, with continuing infections still detected. Additionally, further outbreaks are ongoing in the Democratic Republic of Congo (DRC) and Sierra Leone, with spillover events in surrounding countries [4, 5]. The outbreak in Sierra Leone is caused by a clade IIb lineage A virus, whilst the outbreak in the DRC is caused by a strain belonging to clade I MPXV (clade Ib), which is more virulent in animal models of infection and has a higher reported case fatality rate [6]. Recent detection of clade Ib MPXV cases in numerous countries outside of the African continent, including in Asia, Europe and the Americas [7], together with reports of clade Ib/clade IIb recombinants [8], suggest that future outbreaks are likely and unpredictable [1].

Therapeutic intervention against mpox is very limited. The mpox vaccine, based on the highly attenuated VACV strain modified vaccinia Ankara, is difficult to manufacture at scale and requires two doses to achieve 85% protection [9]. Furthermore, current antiviral drug strategies are lacking, with few antivirals approved for use in humans [9]. Tecovirimat (also known as ST-246 or TPOXX) is the most extensively studied mpox drug so far. Whilst it has no known adverse effects and protects multiple animal species from lethal OPXV challenge [10], data demonstrating clinical efficacy in humans are currently lacking after it failed to meet several endpoints in recent clinical trials [11]. Furthermore, the emergence and transmission of tecovirimat-resistant MPXV strains is a major cause for concern [12]. There is, therefore, an urgent need for the discovery and development of novel antiviral strategies.

Bisbenzimides are a family of DNA-binding compounds that preferentially bind the minor groove of adenine-thymine (AT)-rich dsDNA [13, 14]. Due to their DNA-binding activity, these compounds have shown efficacy both as anti-cancer drugs and as antimicrobials [15–17]. More recently, their potential application as anti-viral compounds has been explored, with a number of bisbenzimide derivatives showing antiviral activity against both human cytomegalovirus (HCMV) [18] and OPXV [19, 20]. In particular, the symmetric bisbenzimide ridinilazole, originally developed as an anti-bacterial agent against *Clostridium difficile* with no adverse effects in human clinical trials [21, 22], has recently been shown to have antiviral activity against HCMV at sub-micromolar concentrations [23]. We, therefore, set out to test whether ridinilazole had similar activity against OPXV and whether it could be repurposed as a much-needed antiviral inhibiting mpox replication.

## Methods

### Cells, viruses and reagents

Human foetal foreskin fibroblasts (HFFF-2, ECACC #86031405), HEK 293 T cells (ATCC #CRL-11268) and BSC-40 (ATCC #CRL-2761) cells were maintained in DMEM (Gibco) supplemented with 10% FBS (Labtech) and 100 U ml^−1^ penicillin plus 100 µg ml^−1^ streptomycin (Pen/Strep; Gibco). Ridinilazole (previously known as SMT19969, CAS number 308362-25-6) was purchased from Cambridge Bioscience. Cytosine arabinoside (AraC) was from MedChem Express (HY-13605). MPXV Clade IIb lineage B.1 (MPXV_CVR_S1; GenBank accession number ON808413) was isolated in 2022 from a patient at the University of Glasgow – Centre for Virus Research (CVR) [24]. VACV Western Reserve (WR, GenBank accession number AY243312) and VACV expressing luciferase (VACV-Luc) (VACV WR engineered to express firefly luciferase from the thymidine kinase locus under the control of the p7.5K early/late promoter [25]) were obtained from Geoffrey L Smith (University of Oxford). VACV WR expressing late proteins F13 and A26 tagged with V5 and FLAG epitopes, respectively, has been previously described [26]. Infection experiments with MPXV were performed in a Biosafety Level 3 laboratory at the Pirbright Institute, Surrey.

### Cell viability assay

Cell viability in the presence of ridinilazole was calculated using the CyQUANT™ MTT Cell Proliferation Assay Kit (Invitrogen) according to the manufacturer’s protocol.

### Viral replication assays

For viral replication assays, cells were seeded at 2.5×10^5^ cells/well in 24-well plates. The following day, cells were infected on ice at the indicated multiplicity of infection (m.o.i.) in 200 µl/well medium. After 60 min the inoculum was removed and the cells washed once with medium to remove unbound virus before 500 µl medium containing 1 µM ridinilazole, or an equivalent volume of DMSO as a control, was added per well and the cells transferred to 37 °C. Virus was harvested at the indicated time points by scraping the cells in their medium and freeze-thawing three times before calculation of viral titres. For VACV, viral titres were calculated by plaque assay on BSC-40 cells as previously described [24]. For MPXV, viral titres were calculated by tissue culture infectious dose 50 (TCID50) assay, according to the Reed and Muench method as previously described [27]. For assays with VACV-Luc, cells were plated 5×10^4^ cells/well in 96-well plates. The following day, the cells were infected at the indicated m.o.i. in the presence of the indicated concentrations of ridinilazole, or DMSO as a control. After the indicated times, the cells were lysed with 100 µl/well passive lysis buffer (Promega) and freeze-thawed prior to luciferase readings. Luciferase activity was then measured with home-made firefly luciferase substrate (20 mM Tricine, 2.67 mM MgSO_4_.7H_2_O, 0.1 mM EDTA, 33.3 mM DTT, 530 µM ATP, 270 µM acetyl coenzyme A, 132 µg ml^−1^ Luciferin (Prolume Ltd.), 5 mM NaOH, 0.26 mM (MgCO_3_)_4_ Mg(OH)_2_.5H_2_O). Fold inductions were calculated by normalizing to a DMSO-treated control. All infection experiments were performed in DMEM supplemented with 2% FBS.

### Reverse transcriptase PCR

For viral gene expression measurements, RNA was extracted from cells using a total RNA purification kit (QIAGEN) according to the manufacturer’s protocol. Five hundred nanogram RNA was used to synthesize cDNA using Superscript III reverse transcriptase (Invitrogen), also according to the manufacturer’s protocol and included a DNase treatment step. cDNA was diluted 1 : 5 in water and 1.5 µl was used as a template for real-time PCR using SYBR® Green PCR master mix (Applied Biosystems) and a QuantStudio 5 (Applied Biosystems) real-time PCR machine. Expression of each gene was normalized to an internal control (*18S-RNA*) to yield a ΔCt. For viral genome measurements, viral DNA was extracted from infected cells using the DNeasy Blood and Tissue Kit (QIAgen) and 1.5 ng was used as a template for qPCR as above using primers recognizing viral gene *F13L* and normalized to a cellular gene (*IFNβ*) to give a ΔCt value. The following primers were used:

*18S RNA*: Fwd 5′-GTAACCCGTTGAACCCCA-3′, Rev 5′-CCATCCAATCGGTAGTAGCG-3′

*IFNβ* Fwd 5′-ACATCCCTGAGGAGATTAAGCA-3′, Rev 5′-GCCAGGAGGTTCTCAACAATAG-3′

*N1L*: Fwd 5′-ATCATCCTAAAGAGTATGGTG-3′, Rev 5′-GCTAGATAGATTGAATCAACC-3′

*F13L*: Fwd 5′-CGATTTACTGTGGCTAGATAC-3′, Rev 5′-ATATCACTTCGGCAAATTTCG-3′

*A26R*: Fwd 5′-ATCTCCCATGTGGTGGAATAC-3′, Rev 5′-GTTGATAGGTTAGAACATCAC-3′

### Immunoblotting

SDS-PAGE and immunoblotting were performed as previously described [24] using the following primary antibodies: rabbit-anti-GAPDH (Proteintech, 0494–1-AP, 1 : 10,000), mouse-anti-FLAG M2 (Sigma, F1804, 1 : 5,000), mouse-anti-V5 (Biorad, MCA1360GA, 1 : 5,000), rabbit-anti-VACV C6 polyclonal serum (1 : 1,000) [28]. Primary antibodies were detected with goat-anti-mouse/rabbit IRdye 680/800 infrared dye secondary antibodies (1 : 10,000) and membranes were imaged using an Odyssey Infrared Imager (LI-COR Biosciences).

## Results and discussion

To determine whether the symmetric bisbenzimide ridinilazole (previously known as SMT19969, CAS number 308362-25-6, Cambridge Bioscience) had antiviral activity against OPXV, we first tested the ability of the compound to inhibit VACV infection (WR strain) in primary HFFF2. To assess the ability of ridinilazole to inhibit VACV spread, HFFF were infected at a low multiplicity (m.o.i.=0.01) in the presence of a range of ridinilazole concentrations and VACV replication was assessed at 24 h post-infection. To facilitate infectivity measurements, we made use of a VACV expressing firefly luciferase (VACV-Luc [25]) and quantified replication by measuring luciferase activity. Ridinilazole strongly inhibited VACV-Luc spread in this cell type, with a half-maximal inhibitory concentration (IC_50_) of 21 nM (Fig. 1a). Consistent with previous observations [23], cell cytotoxicity of ridinilazole as measured by MTT assay was found to be minimal, even at concentrations up to 10 µM (Fig. 1b). Ridinilazole also inhibited VACV spread in a similar assay in HEK293T cells, although not as potently as in the primary fibroblasts (IC_50_=0.91 µM, Fig. 1c). However, ridinilazole showed no antiviral activity when HFFF cells were infected with VACV-Luc at a higher m.o.i. for a shorter time (m.o.i.=2, 8 h), suggesting that it does not inhibit early stages of viral infection, such as entry (Fig. 1d).

**Fig. 1.**
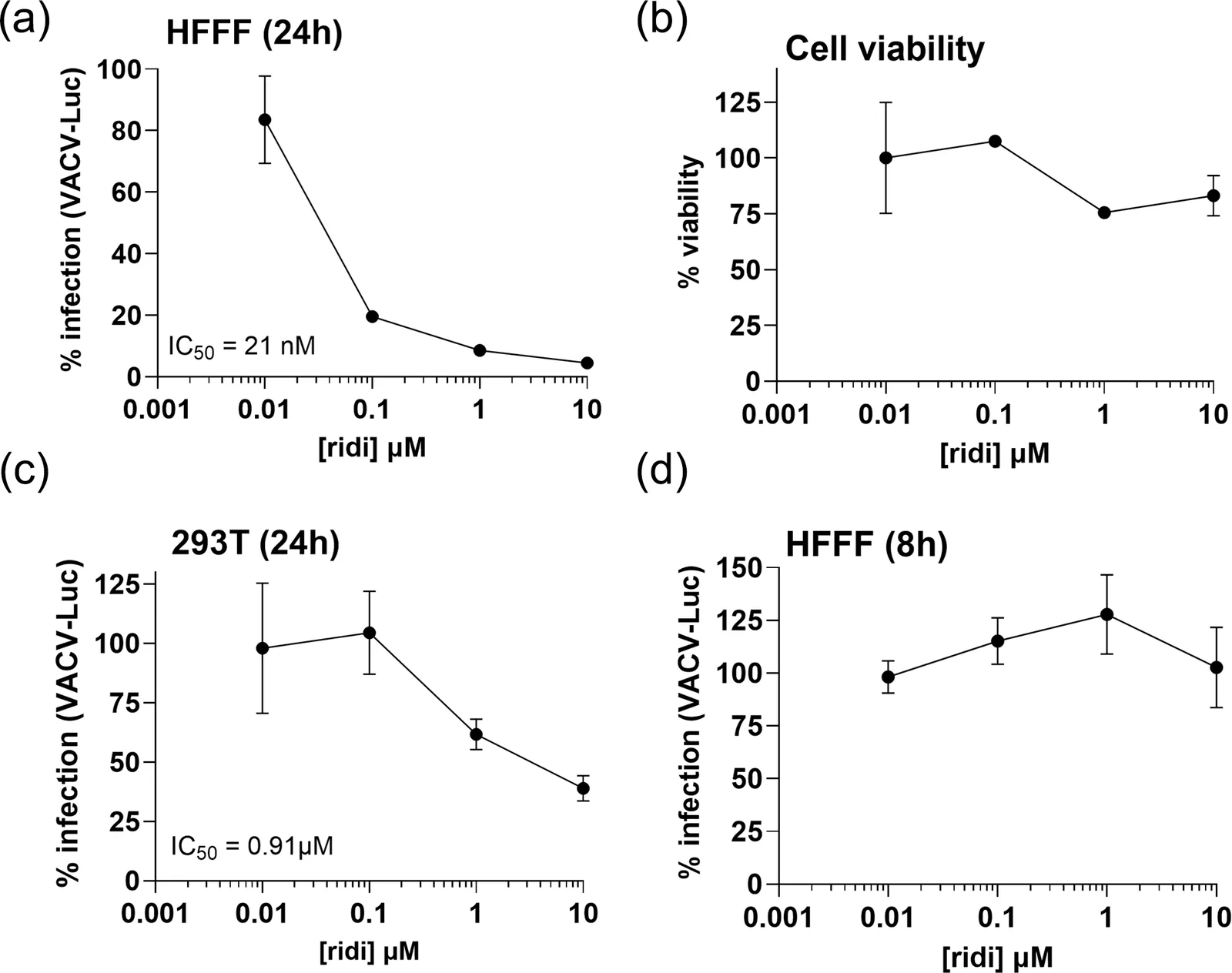
The bisbenzimide compound ridinilazole inhibits VACV spread. (**a**) Percentage infection in HFFF infected with VACV-Luc for 24 h at m.o.i. 0.01 in the presence of the indicated concentrations of ridinilazole (ridi), relative to infected cells treated with an equivalent volume of DMSO. Infection levels in ridinilazole-treated samples were normalized to the corresponding DMSO-treated controls, which were set to 100%. The half-maximal inhibitory concentration (IC_50_, calculated using GraphPad Prism version 10) is indicated in the bottom left. (**b**) Cell viability of HFFF treated for 48 h with the indicated concentrations of ridinilazole (ridi) measured by MTT assay. (**c**) Percentage infection in HEK293T cells infected with VACV-Luc for 24 h at m.o.i. 0.01 in the presence of the indicated concentrations of ridinilazole (ridi), relative to control cells treated with DMSO. The half-maximal inhibitory concentration (IC_50_) is indicated in the bottom left. (**d**) Percentage infection in HFFF infected with VACV-Luc for 8 h at m.o.i. 2 in the presence of the indicated concentrations of ridinilazole (ridi), relative to control cells treated with an equivalent volume of DMSO. Infection levels in ridinilazole-treated samples were normalized to the corresponding DMSO-treated controls, which were set to 100%. Data are presented as mean±sd and are representative of at least three independent repeats, each with three biological replicates.

Next, to confirm the results obtained with VACV-Luc, we infected HFFF with WT VACV WR at both low (m.o.i.=0.5) and high (m.o.i.=3) multiplicity in the presence or absence of 1 µM ridinilazole and measured viral yields from infected cells at 24 h post-infection by plaque assay on BSC-40 cells. Ridinilazole reduced viral yields by at least tenfold following both low (Fig. 2a) and high (Fig. 2b) m.o.i. infection, confirming strong antiviral activity against VACV. Inhibition at high m.o.i. in this assay further confirmed that lack of ridinilazole inhibition observed with VACV-Luc in Fig. 1d was not due to the higher m.o.i. used in this assay, but rather the earlier time point.

**Fig. 2.**
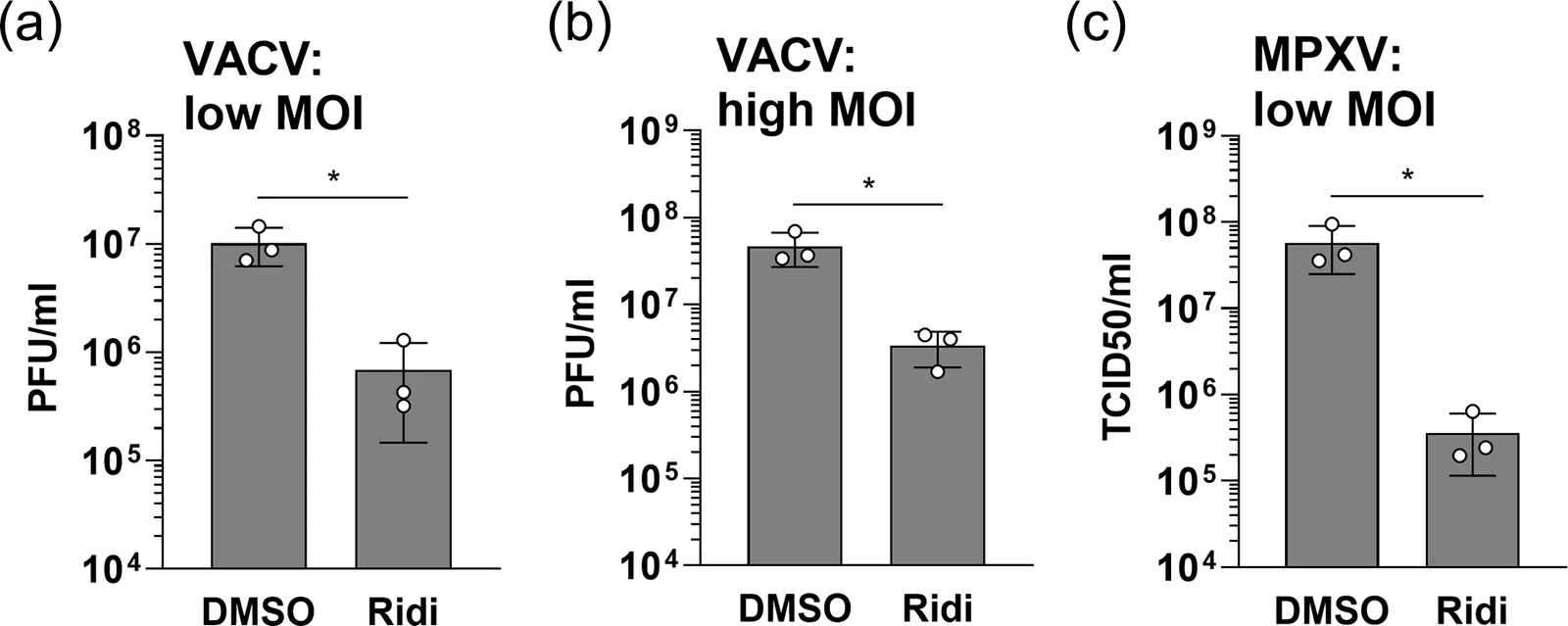
Ridinilazole reduces virus yield and is effective against MPXV (**a, b**) Virus yield from HFFF infected with VACV WR at an m.o.i. of 0.5 (**a**) or m.o.i. 3 (**b**) for 24 h in the presence of 1 µM ridinilazole (ridi) or an equivalent volume of DMSO as a control. VACV titres were quantified by plaque assay on BSC-40 cells (p.f.u. ml^−1^). (**c**) Titres at 48 h from HFFF infected with MPXV at an m.o.i. of 0.05 in the presence of 1 µM ridinilazole (ridi) or an equivalent volume of DMSO as a control. MPXV titres were quantified by tissue culture infectious dose 50 (TCID_50_) assay on BSC-40 cells (TCID_50_ ml^−1^). Data are presented as mean±sd and are representative of at least two independent repeats, each with three biological replicates. Statistical analyses were performed using an unpaired two-sided Student’s t‐test. *P<0.05 (using GraphPad Prism version 10).

Inhibition of VACV replication by ridinilazole prompted us to test for antiviral activity against MPXV. HFFF were infected with MPXV (Clade IIb lineage B.1 2022 global outbreak strain isolated from a patient at the University of Glasgow – CVR, (MPXV_CVR_S1; accession number ON808413) [24] at an m.o.i. 0.01 in the presence or absence of 1 µM ridinilazole and viral yields at 48 h post-infection were assessed by tissue culture infectious dose 50 (TCID50) assay on BSC-40 cells. Ridinilazole inhibited MPXV yields by >150 fold in this spreading assay, demonstrating its potential as a novel antiviral compound against this virus of considerable public health concern in humans (Fig. 2c).

To understand the mechanism of viral replication inhibition exerted by ridinilazole, we assessed the stage of the viral replication cycle at which the compound acts. First, we assessed viral mRNA transcript levels using qRT-PCR [24]. HFFF were infected with VACV at an m.o.i. 5 in the presence or absence of 1 µM ridinilazole and cells were harvested for RNA extraction at 6 h post-infection to determine early viral gene expression (*N1L*) or at 10 h post-infection to assess post-replicative (late) gene expression (*F13L* and *A26R*). Expression of the early *N1L* transcript was equivalent between DMSO-treated and ridinilazole-treated cells, whereas expression of both late viral transcripts was significantly reduced (Fig. 3a). These data were complemented using immunoblotting. HFFF were infected with a recombinant VACV WR expressing late proteins F13 and A26 tagged with V5 and FLAG epitopes, respectively [26], facilitating detection. An antibody against the cellular gene GAPDH was used as an internal control for the amount of protein analysed in each condition. Consistent with qRT-PCR data for viral mRNA transcripts (Fig. 3a), expression of early viral protein C6 was unaffected by ridinilazole at early time points (6 h post-infection), whereas expression of both late proteins F13 and A26 was significantly reduced at 10 and 24 h post-infection (Fig. 3b). Together, these data suggested that ridinilazole inhibited OPXV replication by inhibiting late, but not early, gene expression. Late viral genes encode many proteins that are essential to viral replication, including viral structural proteins and virion assembly and morphogenesis proteins. These findings are consistent with the luciferase assays measured at 8 h post-infection (Fig. 1d) and previous observations of OPXV inhibition with other bisbenzimide compounds [19, 20]. This finding is in contrast to data investigating inhibition of HCMV replication by bisbenzimides, including ridinilazole, where no reduction in either early or late viral protein expression was observed with these compounds, suggesting potentially different modes of action for different viruses and/or different bisbenzimide compounds [18, 23]. Indeed, previous studies found that bisbenzimide Hoechst 33342 inhibited VACV early gene expression as well as late, whereas bisbenzimide Hoechst 33258 inhibited only VACV late gene expression [19]. One limitation of this study is that, because expression was assessed for only two representative late genes, it remains unclear whether ridinilazole selectively inhibits late gene expression or more broadly suppresses the expression of all post-replicative genes, including both intermediate and late genes.

**Fig. 3.**
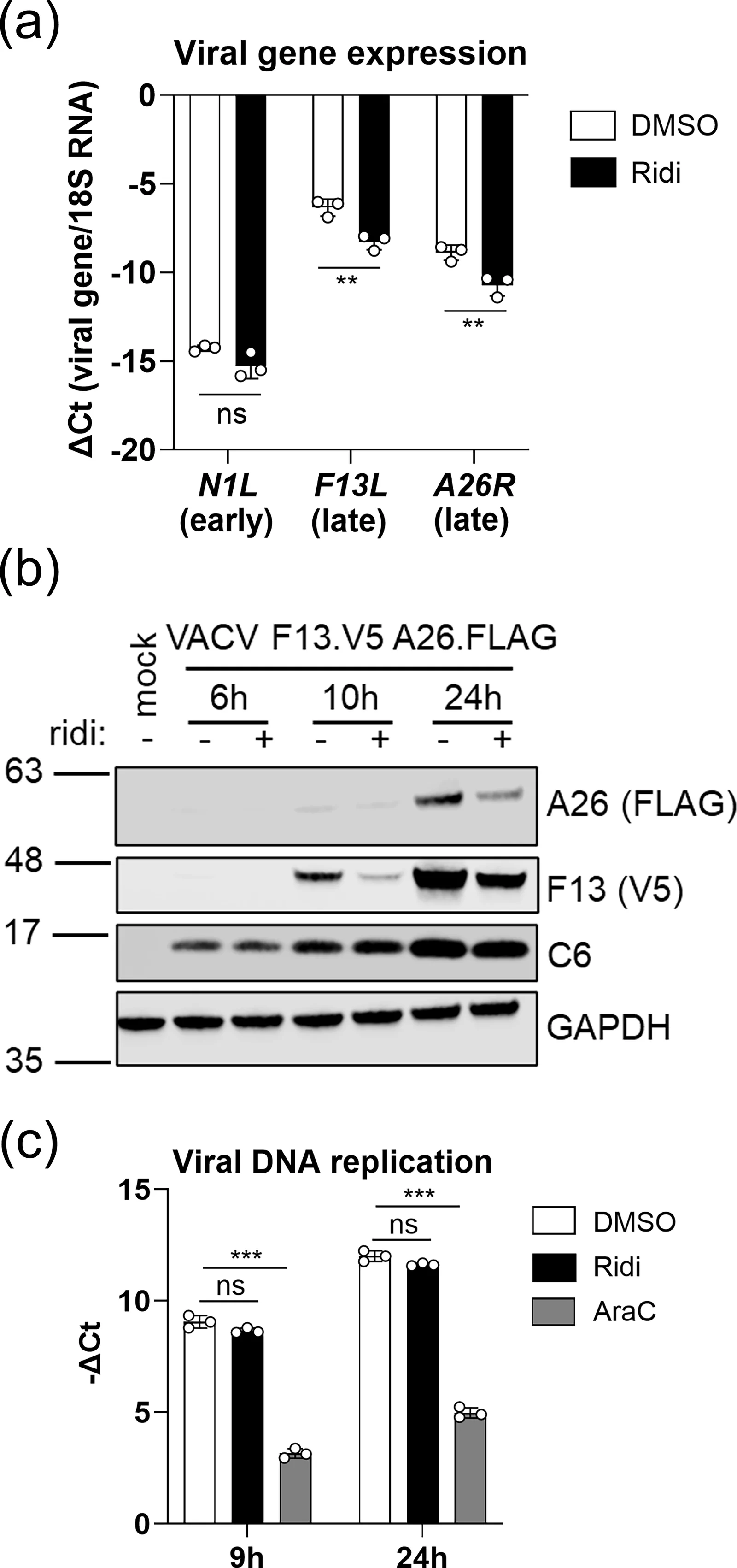
Ridinilazole blocks late gene expression but not genome replication (**a**) Viral gene expression measured by qRT-PCR from HFFF infected with VACV WR at an m.o.i. of 5 in the presence of 1 µM ridinilazole (ridi) or an equivalent volume of DMSO as a control. Early gene *N1L* was measured at 6 h post-infection and post-replicative (late) genes *F13L* and *A26R* were measured at 10 h post-infection. Viral gene expression was normalized to a housekeeping control (*18S RNA*) to give a ΔCt value. (**b**) Immunoblot of lysates from HFFF infected with VACV F13.V5 A26.FLAG at an m.o.i. 5 for the times indicated in the presence of 1 µM ridinilazole (ridi) or an equivalent volume of DMSO as a control. Molecular weight markers are indicated on the left. Lysates were probed for early viral protein C6 and late viral proteins A26 (FLAG-tagged) and F13 (V5-tagged). (**c**) Viral genome measurements by qPCR from HFFF infected with VACV WR at an m.o.i. 5 for the times indicated in the presence of 1 µM ridinilazole (ridi), 40 µg ml^−1^ AraC or DMSO as a control. Viral DNA was measured using primers recognizing viral gene *F13L* and normalized to a cellular gene (*IFNβ*) to give a ΔCt value. Data are presented as mean±sd and are representative of at least two independent repeats, each with three biological replicates. Statistical analyses were performed using an ordinary one-way ANOVA with Tukey’s multiple comparisons test. ns: not significant., **P<0.01, ***P<0.001 (using GraphPad Prism version 10).

Finally, given the reduction in late viral gene expression and the fact that bisbenzimides are DNA-binding compounds, we next assessed whether ridinilazole inhibited viral DNA replication. HFFF were infected with VACV WR at an m.o.i. of 5 in the presence or absence of 1 µM ridinilazole and viral DNA was harvested at 9 and 24 h post-infection, followed by qPCR to detect the viral gene *F13L*. Ct values for *F13L* were normalized to the intronless cellular gene *IFNβ* to yield a ΔCt value. AraC (40 µg ml^−1^), a chain-terminating nucleoside analogue, was used in this assay as a positive control for inhibition of VACV DNA replication. VACV DNA replication was detected at 9 h post-infection and was further increased at 24 h post-infection, and at both time points was greatly reduced by AraC, as expected (Fig. 3c). In contrast, ridinilazole had no effect in this assay. Taken together, these data suggest that whilst ridinilazole inhibits the expression of late OPXV viral genes, this is not due to inhibition of viral replication. How ridinilazole inhibits late gene expression remains to be determined, but it may inhibit transcription from the replicated DNA and/or specifically block transcription from late promoters that are known to consist of a short AT-rich core element [29]. The lack of inhibition of viral DNA replication is consistent with previous observations in HCMV [18, 23]. In contrast, Hoechst 33342 was reported to inhibit both viral DNA replication and early gene expression during VACV infection [19], again highlighting that different bisbenzimide compounds may act through distinct antiviral mechanisms.

In conclusion, the bisbenzimide ridinilazole was an effective antiviral against VACV (IC_50_ of 21 nM in HFFF) and MPXV (<150 fold reduction in titre at 1 µM) with little toxicity both *in vitro* (Fig. 1B, [23]) and in human clinical trials [21, 22], making it a promising candidate for re-purposing as a much-needed novel therapeutic for mpox. Ridinilazole joins an expanding group of compounds identified in recent years as potential antiviral candidates against MPXV, including the antifungal ciclopirox [30], the anti-cancer drug plitidepsin [31], SC144 (an inhibitor of the cellular gp130 pathway) [32], the macropinocytosis inhibitor virapinib [33] and more potent derivatives of brincidofovir [34].

## Abbreviations

AraC: cytosine arabinoside
CVR: Centre for Virus Research
DRC: Democratic Republic of Congo
HCMV: human cytomegalovirus
HFFF-2: human foetal foreskin fibroblasts
MPXV: monkeypox virus
OPXV: orthopoxvirus
RT-PCR: reverse transcriptase PCR
VACV: vaccinia virus
VACV-Luc: VACV expressing luciferase
VARV: variola virus
WR: Western Reserve
